# Statistical and Machine-Learning Analyses in Nutritional Genomics Studies

**DOI:** 10.3390/nu12103140

**Published:** 2020-10-14

**Authors:** Leila Khorraminezhad, Mickael Leclercq, Arnaud Droit, Jean-François Bilodeau, Iwona Rudkowska

**Affiliations:** 1 Endocrinology and Nephrology Unit, CHU de Québec-Laval University Research Center, Quebec (PQ), QC G1V 4G2, Canada; leila.khorraminezhad.1@ulaval.ca (L.K.); mickael.leclercq@crchudequebec.ulaval.ca (M.L.); Arnaud.Droit@crchudequebec.ulaval.ca (A.D.); Jean-Francois.Bilodeau@crchudequebec.ulaval.ca (J.-F.B.); 2 Department of Molecular Medicine, Faculty of Medicine, Laval University, Quebec (PQ), QC G1V 0A6, Canada; 3 Department of Medicine, Faculty of Medicine, Laval University, Quebec (PQ), QC G1V 0A6, Canada; 4 Department of Kinesiology, Faculty of Medicine, Laval University, Quebec (PQ), QC G1V 0A6, Canada

**Keywords:** genomics, multi-OMICS, machine learning, data integration, nutrition

## Abstract

Nutritional compounds may have an influence on different OMICs levels, including genomics, epigenomics, transcriptomics, proteomics, metabolomics, and metagenomics. The integration of OMICs data is challenging but may provide new knowledge to explain the mechanisms involved in the metabolism of nutrients and diseases. Traditional statistical analyses play an important role in description and data association; however, these statistical procedures are not sufficiently enough powered to interpret the large integrated multiple OMICs (multi-OMICS) datasets. Machine learning (ML) approaches can play a major role in the interpretation of multi-OMICS in nutrition research. Specifically, ML can be used for data mining, sample clustering, and classification to produce predictive models and algorithms for integration of multi-OMICs in response to dietary intake. The objective of this review was to investigate the strategies used for the analysis of multi-OMICs data in nutrition studies. Sixteen recent studies aimed to understand the association between dietary intake and multi-OMICs data are summarized. Multivariate analysis in multi-OMICs nutrition studies is used more commonly for analyses. Overall, as nutrition research incorporated multi-OMICs data, the use of novel approaches of analysis such as ML needs to complement the traditional statistical analyses to fully explain the impact of nutrition on health and disease.

## 1. Introduction

In 2003, a new era of genomic studies began after the completion of the human genome project (HGP). Genomics has affected all areas of health sciences and has enabled us to solve many contradictory studies on human health, including nutrition research [1]. The role of various nutrients in gene expression and regulation is considered a key in nutritional sciences. Specifically, nutritional compounds may influence gene expression at different levels including transcription [2], maturing and stability of RNAs, translation process, and post-translational modifications [3,4]. Additionally, the response to the dietary intake depends on the genetic background of an individual which is known as nutrigenetics [5]. For example, Genome-wide Association Studies (GWAS) have reported the contribution of various single-nucleotide polymorphisms (SNPs) in the interaction with nutrients in the development of nutrition-related diseases such as obesity, diabetes, cardiovascular diseases, and hyperlipidemia [6]. Recently, nutrition studies have included the integration of data from other OMICs technologies which refers to the investigation using global analytical technologies, including epigenomics, transcriptomics, proteomics, metabolomics, and microbiomics [7,8]. The interpretation of these OMICs data with machine learning (ML) has made important advances in research [9]. ML is used for sample clustering and the classification of OMICs data as well as to generate a better interpretation of phenotype–genotype relationships [10]. In general, only a few nutrition studies have integrated multi-OMICs data (two or more OMIC datasets) with ML analysis to draw conclusions. There are some challenges to multi-OMICS data together, including 1. the relative importance of changes for given parameters (some genes have an impact even at low expression change while others have a slight biological impact); 2. the relative importance (weight) of data in the analysis in a given pathology (some variables are more critical like glycemia for diabetes); 3. the quality of measurement in different variables used in the multi-OMICs analysis; 4. the proper handling of the missing data in the analysis; 5. the removal of contaminating feature dependence affecting interpretation and ranking (inherently linked clinical data like BMI (body mass index) and weight or composite indexes like the Matsuda index dependent on glucose and insulin); 6. direct correlation (a type of dependence) vs. complex dependence between parameters that cannot be discriminated and correctly interpreted; and 7. strong interactions can mask main feature effects complexifying the interpretation.

The objectives of this review study are 1. to describe the various OMICs techniques; and 2. to examine multi-OMICs analyses in nutrition research, including the supervised and unsupervised ML methods. Furthermore, the advantages and disadvantages of each of the ML methods for the integration of multi-OMICs data are scrutinized. Finally, future directions for integrative analysis in nutrition studies with OMICs (also called nutri-OMICs studies) and its importance in personalized nutrition are discussed.

## 2. OMICs in Nutrition Research

Genomics techniques have been used for many years now in nutrition research. Numerous studies that incorporate one of the genomic technologies, such as genetics, epigenetics, gene expression, proteomics, metabolomics, and microbiomics, can be found in the literature. In this section, each OMICs and associated terms will be defined and described according to nutrition research.

Currently, it is well recognized that individual variability would be associated with the genetic differences in a specific gene. Specifically, a **single-nucleotide polymorphism** (SNP) is a change in the DNA sequence at a particular location in the genome that varies between individuals in a population. In addition, the names of the SNPs are displayed in the format of rsxxxxxx. For example, the association between *Melanocortin 4 receptor* (*MC4R*) locus and nutrient metabolism has been studied in-depth in the literature. Indeed, a population-specific research study reported that the heterozygous G/A genotype of *MC4R* rs2229616 and rs571312 were associated with higher carbohydrate and energy metabolism; however, the homozygous C allele of rs17782313 contributed to higher metabolism of lipids, carbohydrates and energy [11]. Similarly, a systematic review that examined SNP, macronutrients and total energy intake, reported the association between *FTO* SNP rs9939609 and *MC4R* SNP rs17782313 with lower and higher total energy intake [10]. Further, **Genome-Wide Association Studies** (GWASs) which analyze a large number of SNPs (~0.5–1 million) are of interest in nutrition research [12]. For example, individuals with low plasma triglyceride levels following omega-3 fatty acids supplementation have a different GWAS profile compared to those who did not change their plasma triglyceride levels [6]. Since nutrition interacts with genetic factors, studies that investigate the differing genetic effects of a nutrient exposure provide important information on diet recommendations on disease outcomes.

In addition to SNPs, other common genetic variations modify structural DNA by insertions/deletions, translocations, and **copy number variations** (CNVs). For example, a study showed that a high genetic risk score, based on CNVs at three loci, was associated with a higher risk of obesity in Chinese children than a normal CNV [13]. The study also showed that a meat-dominant diet can interact with the CNV at 10q11·22 to increase obesity risk [13]. Therefore, various types of genetic variations may interact with the nutrients to modify the human phenotype and determine dietary requirements.

Nutrient intake is also considered as a key factor to explain the gene–diet interaction through epigenetic mechanisms. **Epigenetic modifications** are changes affecting DNA expression unrelated to DNA sequencing. The main epigenetics modifications include microRNA (miRNA), DNA methylation [11], and histone modifications [10]. Specifically, **miRNAs** are a group of 19–23 nucleotide-long, non-coding, and endogenous RNA molecules. The miRNAs have mediatory roles in RNA silencing and post-translational modifications in gene expression through their presence and/or their levels of expression [14]. For example, the consumption of 1–4 servings/day (250 mL–1 L) milk increased the expression of miR-29b-3p in healthy subjects [15]. Further, **DNA methylation** is defined as adding a methyl group to DNA molecules to change the transcriptional activity of DNA [16]. For instance, a protein-restricted diet may decrease DNA methylation through methionine availability limitations [12]. Lastly, **histone modifications** involve the addition of acetyl (histone acetylation) or methyl (histone methylation), or phosphoryl (histone phosphorylation) groups to histone tails that have key roles in chromatin remodeling of DNA [17]. Dietary bioactive compounds such as organosulfur [18] and curcumin [19] can induce or suppress histone acetylation, respectively. In sum, dietary intake can induce epigenetic changes that modify gene expression and regulation processes.

**Gene expression** is the process by which information from a gene is used in the synthesis of a functional gene product. The importance of analysis of gene expression and transcriptome (the complete set of RNA transcripts that are produced by the genome) in nutrition research is the dynamic nature that can modify metabolic pathways such as carbohydrates, lipids, and energy metabolism [20,21]. For example, after 50 mL/day olive oil for three days, up-regulation was found in *AKAP13* and *USP48* genes related to inflammation and atherosclerosis, respectively [22]. These post-transcriptional changes may eventually alter the function of the proteins in general.

**Nutritional proteomics** is defined as the interaction of food with proteins, which included the effect of nutrients on protein expression, and the interaction of nutrients with proteins in post-translational modifications or small-molecule protein interactions. For example, after a high-fat diet, 50 proteins were differentially expressed between obese and lean mice, and most of those proteins were found in brown adipose tissue [23]. In addition, weight loss resulting from energy restriction (800 kcal/day for eight weeks), caused changes in the number of plasmatic proteins (decrease and increase of 63 and 30 of plasma proteins, respectively) [23]. Overall, proteomics data predict the individual requirements of nutrients based on the protein interactions and enzymatic pathways. Metabolites are small biologically active molecules involved in enzymatic pathways.

**Metabolomics** refers to the monitoring of the levels of metabolites, which are modified by genetics, environment, medication, or dietary intake [24,25]. Specifically, studies have identified that metabolite concentrations have been changed after dietary intakes, such as fruits, red meat, and beef [26,27]. A clinical trial study found that concentrations of tyrosine, lathosterol, and pentadecanoic acid were increased after high-dairy intake (>4 servings/day for six weeks); whereas the levels of 1,5-anhydrosorbitol, myo-inositol, 3-aminoisobutyric acid, and beta-sitosterol were reduced compared to an adequate dairy intake (≤2 servings/day for six weeks) [28]. Furthermore, the consumption of three boiled eggs, 140 g of beef, and fish as sources of choline for a single day enhanced the circulatory plasma levels of choline [27]. The identification of metabolite biomarkers plays a crucial role in the field of nutrition by reflecting the physiological/biological status.

Finally, **microbiomics** is one of the emerging disciplines of OMICs. The gut microbiome communities have been shown complex functions, including the fermentation process, the production of digestive enzymes, as well as the biosynthesis of vitamins and essential amino acids [25]. For instance, low-fat intake (20% of total calories) was associated with increased abundance of *Faecalibacterium* and *Blautia* while high-fat (40% of total calories) diets were associated with the abundance of *Bacteroides* and *Alistipes* phyla [26]. A rat study found that consumption of sourdough-leavened bread (four weeks, 15% *w*/*w*) and a low-protein diet, reduced the abundance of *Alistipes* and *Mucispirillum* in the gut [29]. Moreover, high intake of glucose, sucrose, and fructose found in fruits caused an increase or a reduction of the abundance of *Bifidobacteria* and *Bacteroides*, respectively [30]. Clearly, dietary intakes may have a role in microbial symbiosis to prevent disease or recover more effectively from illness.

## 3. Traditional Statistical Analysis in Nutrition Studies

**Statistical analyses** include organization, description, correlations, the discovery of the interaction between factors, and interpretation of data [31]. Traditional nutritional data analysis consists of two steps: 1, converting data into analytical variables; and 2. selecting an appropriate statistical test according to the purpose of the study, study design, and nature of the data (continuous and categorical) [32]. For instance, *t*-tests (paired *t*-test, independent sample *t*-test), analysis of variance (ANOVA) or analysis of covariance (ANCOVA) and correlation (Pearson and Spearman) are considered the common analytical methods for continuous variables (such as BMI) [33]. *t*-tests are very easy and interpretable tests that compare differences between two groups; however, *t*-tests are used for sample size less than 30 to have enough reliability and accuracy [34]. Moreover, multiple comparisons are impossible through the paired data *t*-test [35]. Unlike *t*-tests, the **ANOVA** test is used to compare differences between multiple groups; however, using a one-way ANOVA may be difficult to determine which group varies from other groups [36]. For instance, differences in bone density between three visfatin genotypes (GG, GT, TT) were assessed by ANOVA test in a nutrigenomics study on obese and overweight healthy adults [37]. However, a *t*-test was used to examine the differences in lipid profile, inflammatory parameters between two vitamin D-binding protein (polymorphism, rs4588) genotypes (CC, AC + AA) [38]. Furthermore, s two-way ANOVA is used to measure the effect of two different categorical variables on one continuous variable. For example, an animal study indicated that interaction of categorical variables, including high-protein (45% protein) and high physical activity was associated with reduced total cholesterol and low-density lipoprotein among mice [39]. Besides, **ANCOVA** has efficiency and power to find and estimate the interactions and the ability to deal with the measurement errors in the covariates, although ANCOVA is inappropriate for large data [40].

In addition, **correlation** tests such as bivariate correlation (measure the association between two continuous variables) and partial correlation (determine the relationship between two continuous variables while adjusting for one or more continuous variables), are an association test which does not imply cause and effect relationship, and may not determine which variable is considered to have the most influence [41]. Further, **Chi-square** and **regression** (logistic regression and multinomial regression) are recognized as the major analyses for categorical variables [42]. The **Chi-square** is sensitive to sample size (*n* < 20) since by increasing the sample size the difference becomes smaller and less precise [43]. The **logistic regression** is a method to predict the association between binary dependent variables and one or more independent variables. The logistic regression may provide perfect algorithms to avoid overfitting but this method is not flexible enough for multiple data (such as multi-OMICs data) with a large number of variables and complex associations [44]. The **multinomial regression** is a predictive analysis used when the dependent variable is nominal (two or more levels). Moreover, the **General Linear Model** (**GLM**) is a multivariate regression method with the purpose to compare the association between dependent variables and continuous/categorical independent variables. Moreover, the dependent variable must have a normal distribution in general linear regression. For these reasons, the **generalized linear mixed model** (**GLMM**) is preferred for the non-normal distribution of residues since it allows for more options in the type of distribution used to fit the model. For example, a GLM was used to compare the variation in sugar balance between individuals with acceptable sugar (≤10% of total energy) and excess sugar (>10% of total energy) through different food groups and subgroups [45]. Overall, regression analysis is commonly used to examine the association between two or more variables (categorical or continuous) in nutrition research.

## 4. Machine Learning in Nutrition Studies

Based on the large volume of data in the nutri-OMICs studies, ML may be the best approach to identify the association between nutrient intake and OMICs pathways. **ML** is one of the major fields of artificial intelligence that provides powerful computer systems to characterize, learn, and perform algorithms and models with unique precision. A major advantage of ML is the ability to learn and make algorithms without human intervention. In addition, the accuracy of ML analysis improves with the addition of training data. Further, the analysis should be conducted according to the hypotheses of the study to minimize the disadvantages of the procedure.

In the **process of multi-OMICs analyses**, first, the features (variables) with the greatest contribution to the prediction output are selected. Second, the selected features are analyzed by different methods of ML to integrate all of them to make a prediction model. In the next section, this review will examine the different methods of data integration in supervised and unsupervised ML.

### 4.1. Supervised Machine Learning

**Supervised ML** considers the learning function that provided output based on input data through the training data including a set of training examples [46]. The feature selection process is defined to select a subset of relevant features (variables) to predict a model construction. The feature selection influences the performance of the model by reducing overfitting and improving accuracy [47]. For example, in a human study of a calorie-restricted diet, the selection was performed to find the most related features to insulin sensitivity, such as metabolites, gut microbiota, food groups, and the nutrients [48]. Overfitting is a modeling error that occurs when functions are fitted to a limited or a particular set of data. The aim of integrative models and algorithms is to find and select relevant variables that can accurately predict and estimate the risk of disease with the simpler model [48].

#### 4.1.1. Data Preparation

The goal of supervised ML is to produce predictive models and extraction of algorithms by technical data mining. In the nutrition context, **data mining** is characterized by the extraction of patterns and identification of key features (markers) to find correlations within the genomics, proteomics, metabolomics, and gut microbiota data sets. One of the major data mining methods to study biological networks is **Weighted Correlation Network Analysis** (**WGCNA**) that is used to perform pairwise correlations between variables [49]. In addition, various ML feature selection algorithms exist to exclude uninformative features from OMICs data.

#### 4.1.2. Classification Methods in Supervised Machine Learning

The supervised ML for classification includes **Naïve Bayes** (**NB**), **Support Vector Machines** (**SVM**), **k-Nearest Neighbor’s algorithm** (**k-NN**), and **Random Forest** (**RF**). One of the simplest algorithms is the **NB** which is considered as a probabilistic classifier based on different attributes in data. In a childhood study using the NB method, toddlers’ anthropometric status was categorized into three groups with different accuracy including 88% of the weight-for-age index; 64% for the height for age index; and 68% for the weight-for-height index [50]. Consequently, based on anthropometric standards, the nutrition status of a toddler is measured. The **SVM** is a supervised classifier to analyze data sets related to learning algorithms that are used for classification and regression analysis [51]. The SVM method was used to predict a model in the relationship between metabolized energy and dietary chemical profiles (crude protein, ether extract, crude fiber, and starch (g/kg)) [52]. The **k-NN** and **RF** models are recognized as two methods of regression trees in dietary pattern extraction [53]. The **k-NN** is a supervised method for estimation and pattern recognition which classifies cases based on the number of nearest neighbors (k) to a majority features space. The **RF** is used to perform classification and regression using a multitude of decision trees at training time [54]. Overall, the supervised classification will be used to illustrate the differences between classifiers. The second category of supervised ML is regression. Many regression algorithms are able to perform classifications [55].

#### 4.1.3. Regression Method in Supervised Machine Learning

**Linear regression** is used to predict the independent variable value based on the dependent variable. Furthermore, the linear regression is used for continuous variables and may not be appropriate enough for data with non-linear associations or a large number of variables (*n* > 100) [56]. Overall, regression supervised ML makes a model to explain the association between feature data set and continuous dependent variables.

### 4.2. Unsupervised Machine Learning

The **unsupervised ML** goal is to discover natural and hidden patterns or distributions in the data, without output variables and previous training dataset. In addition, unsupervised ML needs an external evaluation to be sure that the results are meaningful [57]. There are two unsupervised ML methods. First, the **clustering method**, as the most important unsupervised method, is performed to determine inherent clusters based on the natural structure and unlabeled data. Secondly, the **K-means clustering** (**K-cluster**) is one of the simplest and most used clustering methods. In the K-cluster method, a cluster is included in the collection of data with specific similarities. The K-cluster may not appropriate enough for small sizes and density of clusters; however, this method is well-scaled for large data sets and is considered the fastest technique of clustering [58]. Overall, unsupervised learning is mainly used to find patterns and clustering data set which are not known before in the dataset.

### 4.3. Multivariate Analysis

ML also encompasses **multivariate analyses** (**MVA**) by nature, but supplementary statistical approaches have been developed to estimate the association between more than two variables (data are more than one type of measurement or observation) as well as to find patterns and associations between outcome variables [59]. Still, ML and MVA are different since there are supervised learning techniques in ML outside the regular MVA. MVA is an extension of bivariate regression but considers two or more independent variables and has the advantage to reduce the dimensionality (reduce the number of features) of a data set when numerous features exist and are uninformative. MVA is divided into three categorizations, which mainly includes factor analysis (divided data to smaller groups based on similar response patterns), cluster analysis (classification of a large data set to different groups based on similar characteristics), and regression analysis (computing the association between an independent variable and one or more dependent variables). However, MVA analysis requires a large dataset; otherwise, the analysis becomes statistically meaningless due to the high standard error [49].

#### 4.3.1. Supervised Multivariate Analysis

**Supervised MVA** includes classification and regression analysis. **Orthogonal Projections to Latent Structures Discriminant Analysis** (**OPLS**-**DA**) is a powerful modeling tool to identify the difference between two groups and the variable with larger discriminatory power [60]. Whereas, **partial least squares discriminant analysis** (**PLS-DA**) is defined as an MVA extension of a paired *t*-test and used when data has different levels such as multi-OMICs data [60]. In a nutri-OMICs study, to compare the metabolite profile after consumption of 10.4 g/day arabinoxylan-oligosaccharides, OPLS-DA was used [61]. Furthermore, **partial least square regression** (**PLSR**) is considered a linear regression MVA with the ability to analyze large redundant features to construct predictive models [62].

#### 4.3.2. Unsupervised Multivariate Analysis

**Unsupervised MVA** includes factor and clustering analysis. **Multivariate analysis of variance** (**MANOVA**) is an MVA analysis and is very similar to ANOVA, which used to examine the association between two or more dependent variables and one or more independent variables. For example, **Principal Component Analysis** (**PCA**) as a factor analysis method, have been used to investigate dietary patterns in nutrition studies [63]. The PCA analyzes the correlation and covariance between quantitative variables. The **Principal Coordinate Analysis** (**PCoA**) is a method to explore the similarities or dissimilarities between variables. For instance, by using PCoA analysis the dissimilarities and sample clusters of gut microbiota were visualized after consumption of high-fat (60% of total energy) and low-fat diets (10% of total energy) [64]. Overall, all MVA are used to identify the correlation or covariance between multiple phenotypes of disease and OMICs to provide enhanced biological pathway models [65].

**Figure 1:**
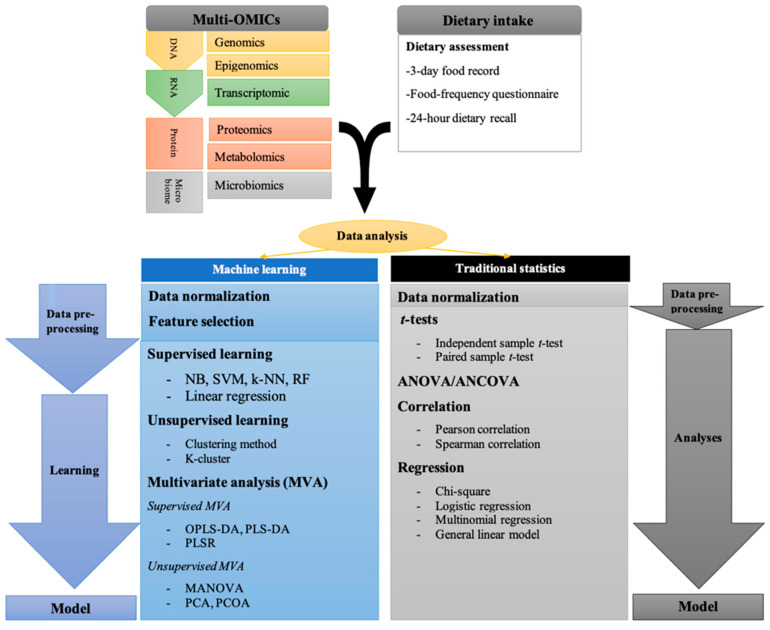
Traditional statistical, and machine analysis (supervised, unsupervised machine learning and multivariate analysis) were used in nutri-OMICs studies. Analysis of variance, ANOVA; k-nearest neighbor’s algorithm, k-NN; Support vector machine, SVM; Regression random forest, RF; Naïve ayes, NB; Partial least-squares regression, PLSR; Orthogonal projections to latent structures discriminant analysis, OPLS-DA; Partial least squares discriminant analysis, PLS-DA; Principle component analysis, PCA; Principal coordinate analysis, PCoA; Multivariate analysis of variance, MANOVA.

Overall, ML has a crucial role in multi-OMICs studies through feature selection and data mining. Further, the regression and classification methods are used to find the relationship and interaction between features. Studies have used ML analysis through web servers, dedicated software, and tools to integrate OMICs data in various disorders [66,67,68]. Furthermore, **pathway analysis** is used to associate OMICs data with the dietary intake (food frequency questionnaire, three-day food records, and 24 h dietary recalls) in nutri-OMICs studies. Therefore, ML has the capacity to predict more complex and complete models compared to classical statistical methods to explain the association between multi-OMICs with dietary variables (Figure 1). Nutrigenomics researchers are beginning to incorporate multi-OMICs analytical approaches and using ML seems necessary due to the complexity as well as advances in molecular nutrition. In the next section, the ML analysis performed in nutritional genomics studies is reviewed.

## 5. Multi-OMICs Studies in Nutrition Research

As described above, studies have examined the link between OMICs and nutrients in predicting individual responses to dietary intake. Now, given the accessibility of multi-OMICs, nutrition scientists can enhance their research by incorporating various OMICs data in their studies. The objectives of nutri-OMICs studies are to report: 1. the variations in nutrients and OMICs; and/or 2. the correlations and interactions between the variables or factors to draw conclusions, from the observational or experimental studies. The requirement for advanced analytical strategies is needed to adapt to the recent access to OMICs and multi-OMICs data [69]. To our knowledge, only 16 studies have integrated nutrition data (such as food frequency or dietary recall) with multi-OMICs data (see Table 1: Multi-OMICs in nutrition research) to explain the molecular mechanisms of diets and food supplements on health.

MVAs are the most popular approaches used in nutri-OMICs studies. Supervised MVA such as OPLS-DA, PLS-DA, and PLSR are used for data analyses in several nutrition studies. A study showed that vegetarians compared with omnivorous adults have: 1. lower branched-chain amino acid (BCAA) concentrations using OPLS-DA to compare the metabolome; and 2. a higher abundance of *Prevotella* and a decrease in abundance of *Bacteroides* using PCoA to compare the microbiota [73]. In another study by using OPLS-DA, a reduction in fasting insulin and homeostatic model assessment of insulin resistance (HOMA-IR) were correlated to an increase in the abundance of gut bacteria such as *Actinobacteria*, *Bifidobacteriaceae*, and *Bifidobacterium* after consumption of arabinoxylan-oligosaccharides [61]. Furthermore, using PLS-DA, a recent animal study found that intake of dietary inulin compared to cellulose, reduced the concentration of BCAA, L-valine, and L-isoleucine together with an increased level of indole-3-propionic acid and an increase in the abundance of *Firmicutes* and *Bacteroidetes* [71]. Cross-correlation using PLS-DA was conducted in a study on mice which has reported that a high-fat diet (45% kcal from fat) supplemented by high-amylose-maize resistant starch type 2 changed gene expression of fatty acid metabolism and hepatic metabolism. The latter coincided with an increase in the abundance of *Tenericutes*, *Bacteroidetes*, and *Verrucomicrobia,* and a reduction in the abundance of *Proteobacteria* and *Firmicutes* phyla [78]. Lastly, for supervised MVA using PLSR, a calorie restriction diet (low-fat (25% of total calories) and high-protein (35% of total calories)) was shown to decrease serum BCAA levels together with enhanced insulin sensitivity in overweight or obese participants [48].

Unsupervised MVA analyses such as PCA and PCoA are also often used in nutrition studies. For example, with PCA, a mouse study has found that upregulation of iso-citrate dehydrogenase, lipid metabolism, and adenosine triphosphate (ATP) turnover were related to anti-obesity effects of different types of coffee (caffeinated coffee, decaffeinated coffee, and green unroasted coffee) when combined with a high-fat diet (60% of total calories) [81]. Similarly, a PCA analysis between different groups of proteins showed that the down-regulation of proteins related to energy metabolism was associated with an increase in the abundance of *Bacteroidetes* and *Firmicutes* after an eight week intervention with a cafeteria diet in rats (49% fat of total energy) [74]. Also, an animal study with an American diet (50% carbohydrate, 15% protein, and 35% fat, for nine weeks) compared to a normal diet (25–30% fat, for nine weeks), demonstrated that the amount butyrate in the stool (the difference between metabolites in two diets measured by PCA) was positively correlated (by correlation test and network analysis) with the abundance of butyrate-producing bacteria, *Oscillospira* and *Ruminococcus* (differences between microbiota between two diets by PCoA) [77]. Furthermore, a greater impact of gut microbiome compared to dietary intake (medium-fat and carbohydrate meal) was found on postprandial lipemia using a PCA analysis [70]. Further, PCoA analyses were used to compare children with Prader–Willi syndrome and healthy children, show an increase in gut *Bifidobacteria* and short-chain fatty acid (SCFA) production (acetate) after a diet containing whole grains, traditional medicinal foods, and probiotics [79]. Also using PCoA analysis, a study found an increase in gene expression for liver enzymes after consumption of a high-fat diet (60% of total calories) compared to a low-fat diet (10% of total calories), that was associated with gut microbiota composition in *Lachnospiraceae*, *Ruminococcaceae*, *Streptococcaceae*, and *Lactobacillaceae* [64]. Finally, for unsupervised MVA studies, the PCoA and regression-based analysis were used to show that lower postprandial glycemic responses are correlated to alterations in the gut abundance of *Proteobacteria* and *Enterobacteriaceae* [80].

Supervised and unsupervised MVA based on mixDIABLO (Data Integration Analysis for Biomarker discovery using Latent variable approaches for ‘Omics studies) demonstrated that a low-carbohydrate diet (<30 carbohydrate gr/day for seven days) up-regulated the genes in various metabolic pathways, including the peroxisome proliferator-activated receptor (PPAR) signaling and fatty acids degradation in the blood circulation [76]. Concurrently, serum concentrations of β-hydroxybutyrate and folate-producing bacteria (*Streptococcus* and *Lactococcus*) were increased by the low-sugar diet [76].

Other ML methods have also been incorporated in nutrition studies. A generational study (parents and children as participants) on healthy participants demonstrated that serum lipid profile and total serum carotenoids were closely related to the expression of genes associated with metabolism and inflammatory pathways using weighted correlation network analysis (WGCNA) [72]. Further, the consumption of plant-derived nutrients (vitamins and phytochemicals) was correlated with WGCNA to reduced plasma taurodeoxycholate among individuals with a low abundance of *Ruminococcaceae* using PCoA analyses (4). Lastly, a study by Piening et al., 2018 [75] used the RF to demonstrate that a high-caloric diet (increase 880 kcal per day for 30 days) deregulated the BCAA metabolism in conjunction with the activation of inflammatory signatures including C-reactive protein and the abundance of the *Verrucomicrobiaceae* family. In sum, the use of ML techniques contributes to a better understanding of the molecular mechanisms of diets and foods on nutritional health.

## 6. Conclusions and Future Directions

The growing improvements in laboratory techniques have increased the complexity and a large number of generated data. Furthermore, multi-OMICs studies compared to single OMICs have provided new information to describe the role of nutrients in molecular pathways using together either gene protein, metabolites and/or gut bacteria. However, there are a limited number of multi-OMICs nutrition studies that used supervised and unsupervised ML so far; nonetheless, the number of studies is increasing due to advances and researcher familiarization with ML. Various traditional statistical methods and ML methodologies have been used to integrate nutri-OMICs data. These methodologies are complementary, and the selection of the appropriate ML approach also depends on the coherent conclusion draw by ML with the vast knowledge in biology and nutrition, the distribution, type of data, and the aims of the study. Yet, MVA especially PCA, PCoA, OPLS-DA, and PLS-DA are the most popular approaches used in nutri-OMICs studies (Table 1). The analysis of multiple layers of OMICs presents a challenge that ML can realistically tackle in a less time-consuming manner than with traditional statistical approaches. The integration of OMICs may increase progress in personalized nutrition compared to the association between dietary intake and single OMICs category alone. Further studies are needed to determine the most accurate algorithms and analytical approaches in multi-OMICs studies. Nutrition studies should be performed to also compare the accuracy of ML versus traditional statistical analyses for validation. Overall, the integration of multi-OMICs data in nutrition research through ML techniques compared to conventional statistical analysis methods may provide a robust contribution to the impact of nutrition on health and diseases.

## Figures and Tables

**Table 1:** Human and animal studies using multi-OMICs approaches for the investigation of dietary intake on health and disease states.

References	Type of Study	Population Omics	Methodology	Main Analysis Strategy	Main Finding
Berry SE. et al., 2020 [70]	Cohort study (multi-national study)	*N* = 1002 healthy adults (UK) *N* = 100 healthy adults (USA)	Nutrition assessment: Food frequency questionnaires Biochemical measurement: GCM, ADVIA chemistry triglyceride and glucose oxidase method Genomics: Illumina Infinium HumanHap610 Microbiomics: 16S rRNA and arrays	*Multilinear* ANOVA (Hierarchical Bayes models) Random forest regression (Unsupervised ML) PCA (Unsupervised ML)	Medium-fat and -carbohydrate lunch showed a less impact on postprandial lipemia compared to gut microbiome while genetic had a modest influence on glycemic and lipid profile.
Wu W. et al., 2020 [71]	Animal study	*N* = 12 (pigs) 6 pigs (case): a maize-soybean meal diet containing 5% a high-fermentable fiber (Inulin) 6 pigs (control): a 5% low-fermentable fiber (cellulose) control	Metabolomics: GC-TOF-MS method Microbiomics: 16S and whole metagenome	PERMANOVA (Multivariate analysis- unsupervised ML) PLS-DA (Multivariate analysis-supervised ML) Integrative analysis: mixOmics package of R software	Inulin intake has effects on the increasing the diversity of microbiota composition in the cecum along with a decrease of the circulating of metabolites including branched-chain amino acids, L-valine, L-isoleucine and an increase in the level of indole-3-propionic acid.
Sundekilde U.K. et al., 2020 [64]	Animal study	*N* = 20 mice (C57Bl/6J), males, 6-week old Group 1: 60% fat (high-fat diet; HFD) Group 2: 10% fat (low-fat diet; LFD)	Genomics: (RNA extraction, Illumina, Qiagen) Metabolomics: NMR Spectroscopy, LC-MS analysis on urine and plasma, LC-MS analysis on tissue samples, GC-MS analysis Microbiomics: 16S and whole metagenome	PCoA (Multivariate analysis-unsupervised ML)	Increase in malate, succinate and oxaloacetate levels were associated to down-regulation of gene expression of malate dehydrogenase together with gut microbiota enrichment (Lachnospiraceae, Ruminococcaceae, Streptococcaceae, Lactobacillaceae) in HFD compared to LFD mice.
Tremblay B.L. et al., 2020 [72]	Observational study	*N* = 48 healthy Parents = 22, age, 42.3 year Children = 26, age, 11.3 year	Nutrition assessment: carotenoid measurements High-performance liquid chromatograph (HPLC), ChemStation software Biochemical measurement: Enzymatic assays, Friedewald formula, the rocket immunoelectrophoretic method Genomics: DNA and RNA extraction (microarray platform, Illumina) Epigenomics: Methylation; Infinium Human Methylation 450 array	One-Sample Wilcoxon Signed Rank Test Linear regression Clustering method (Unsupervised ML) Correlation based analyses WGCNA (Data mining method)	Genes expression in lipid metabolism and inflammatory pathways together with DNA methylation have a mediatory role in the association between total carotenoids and lipid profile in plasma.
Benitez-Paez A. et al., 2019 [61]	Randomized crossover study	*N* = 15 overweight subjects Duration: 4-weeks for each phase 10.4 g/day AXOS (Arabinoxylan-oligosaccharides)	Biochemical measurement: plasma and fecal bile acids (LC-MS/MS) fecal lipid species (LC-MS/HRMS) Microbiomics: 16S and whole metagenome Metabolomics: NMR Spectroscopy	Paired and one-sided *t*-test or Wilcox signed-rank test Logistic regression model PCA (Unsupervised ML) OPLS-DA (supervised ML)	Increase in the abundance of Actinobacteria, Bifidobacteriaceae, Bifidobacterium and change the host metabolism including glucose homeostasis (reduction in fasting insulin and HOMA-IR) after consumption of AXOS.
Wang F. et al., 2019 [73]	Preliminary study	*N* = 36, age = 28.1 Duration of study = 6 months Vegan = 12 Lacto-ovo vegetarian = 12 Omnivorous = 12	Nutrition assessment: 3-day food records Metabolomics: Gas chromatography coupled to time-of-flight mass spectrometry system Metatranscriptomic: Illumina HiSeq 4000, KEGG, using BLASTP Microbiomics: 16S and whole metagenome	Chi-square and *t*-test for PCoA (Multivariate analysis-unsupervised ML) Clustering method (Unsupervised ML)	Decrease concentrations of BCAAs, the abundance of Prevotella and Bacteroides were increased and decreased, respectively, among vegetarians compared with omnivores.
Tang Z.Z. et al., 2019 [4]	Cross-sectional study	*N* = 150 healthy (55 M, 95 F) Age 18–50 year	Nutrition assessment: 3-day food records and food frequency questionnaires Metabolomics: (untargeted LC-MS) Microbiomics: 16S and whole metagenome sequencing from stool	Correlation based analyses Sparse Linear Log-Contrast Model (Supervised ML) Network analysis-WGCNA) (Data mining method)	Mediatory role of Ruminococcaceae in the association of plant-derived food and artificial sweeteners with bile acids in stool.
Guirro M. et al., 2018 [74]	Animal study	*N* = 24 male Sprague-Dawley rats (8-week old) Duration: 9-week Two groups: *N* = 12, cafeteria (CAF) *N* = 12 standard chow (STD) After intervention (8-week): Each diet group supplemented: 1. Low-fat condensed milk (*n* = 6) 2. Hesperidin dissolved with low-fat condensed milk (*n* = 6)	Metaproteomics: NanoLC-(Orbitrap) MS/MS analysis Microbiomics: 16S and whole metagenome sequencing from stool	Univariate statistical analysis (Student’s *t*-test) PCA (Multivariate analysis-unsupervised ML)	Increase the abundance of Bacteroidetes and Firmicutes, which are related to down-regulation of proteins in energy metabolism pathways such as the tricarboxylic acid cycle or ATP-binding pathways after CAF diet.
Dao M.C. et al., 2018 [48]	Cohort study	27 F (24), M (3) overweight or obese adults 6-week calorie restriction (CR): 1200 kcal/day (F) 1500 kcal/day (M)	Nutrition assessment: 7-day food records Genomics: DNA (microarray platform, Illumina) Transcriptomics: Microarray platform (Illumina) Metabolomics: Gas chromatography system (GC–MS) and H-NMR Spectroscopy	Nutrition analysis (Profile Dossier v3 & Profile Dossier x029) PLSR (Supervised ML)	Increase in insulin sensitivity and BCAA after CR associated with gut microbiota, metabolomics and adipose tissue genes in both genders.
Piening B.D. et al., 2018 [75]	Case-control study	13 Insulin resistance (IR) participants F (7), M (6)-age: 58, BMI: 30.5 10 Insulin sensitivity (IS) participants BMI 25–35 kg/m^2^, F (7), M (3)-age: 56, BMI: 28.5 First, hypercaloric diet for a period of 30-day (increase 880 kcal per day) Second, iso-caloric diet for 7 days Third, a caloric-restricted (CR) diet 60-day period	Proteomics: Untargeted liquid chromatography (LC-MS) Metabolomics: Untargeted LC-MS Microbiomics: 16S and whole metagenome sequencing from stool)	Correlation and regression-based analyses Random forest classification (Supervised ML) Interaction model: generalized linear models (GLMs)	Dysregulation of antimicrobial response (CAMP, LFT, and defensins) was reflected in proteome and circulating cytokines in IR compared to IS participants.
Mardinoglu A. et al., 2018 [76]	Short term intervention study	*N* = 10 (NAFLD), F (2)–M (8) BMI: 34.1, age: 47 Intervention: Isocaloric, low-carbohydrate diet (23–30 g/day) with increased protein (24% of total energy)-14 days	Transcriptomics: Microarray platform (Illumina) Metabolomics: Untargeted Analyses UPLC/MS/MS-GC/MS	Correlation based analyses Linear mixed effect model The multivariate analysis based on mix DIABLO (Supervised and unsupervised ML)	Increase in serum concentration of β-hydroxybutyrate concentrations, mitochondrial β-oxidation, and folate producing Streptococcus and serum folate after intervention.
Ishii C. et al., 2018 [77]	Case-control study	Mice (C57BL/6J) 52 weeks: Normal diet (*n* = 6) American diet (*n* = 5)	Metabolomics: (Ultrafree MC) Metagenomics: (microarray platform, Affymetrix)	Correlation based analysis PICRUST software analyses (Supervised and unsupervised ML)	Abundance of genes associated with butyrate metabolism is positively correlated with butyrate producing bacteria (Oscillospira and Ruminococcus).
Kieffer D. et al., 2016 [78]	Animal study	45% kcal from fat + high-amylose-maize resistant starch type 2 (HAMRS2), Case, *N* = 14 5-week old male (C57BL/6J mice) Digestible starch, Control, *N* = 15 5-week old male C57BL/6J mice Duration: 5 weeks	Metabolomics: Untargeted GC-MS Microbiomics: 16S and whole metagenome sequencing from stool, analyzed by QIIME	Correlation and *t*-test-based analyses PLS-DA (Multivariate analysis- supervised ML)	Changes in hepatic metabolism and gene expression related to fatty acids metabolism together with increases in Tenericutes, Bacteroidetes, Verrucomicrobia and decrease in Proteobacteria and Firmicutes phyla after HAMRS2 diet.
Zhang C. et al., 2015 [79]	Case-control study	*N* = 17, Prader–Willi syndrome (PWS), duration 90 day *N* = 21, heathy obese, duration 30 days Intervention: WTP diet (whole grains, traditional Chinese medicinal foods, and prebiotics)	Nutrition assessment: 24-h dietary record Biochemical analysis: Serum glucose, CRP, total cholesterol, triglycerides, free fatty acids, ALT and AST (automatic biochemical analyzer (ADVIA^®^ 1800 Clinical Chemistry System), Insulin (immunochemiluminometric assays), HbAlc (HPLC) Metabolomics: NMR Spectroscopy Microbiomics: 16S and whole metagenome sequencing from stool	Wilcoxon matched-pairs signed rank test (two-tailed) Independent Mann–Whitney U test (two-tailed) OPLS-DA	Balance of gut microbiota composition which contributes to the alleviation of metabolic deterioration in obesity among children with Prader–Willi syndrome after consumption of a diet rich in fermentable carbohydrates. Children genetically obese with Prader–Willi syndrome shared a similar dysbiosis in their gut microbiota with those having diet-related obesity.
Zeevi D. et al., 2015 [80]	Cohort study	*N* = 800 healthy, F (60%)–M (40%) 54% Overweight 22% Obese	Nutrition assessment: Dietary habits (www.personalnutrition.org) Biochemical analysis: Glucose was measured for 7 days using the iPro2 CGM with Enlite sensors Genomics: DNA extraction (microarray platform, Illumina) Microbiomics: 16S and whole metagenome sequencing from stool	Correlation and regression-based analyses PCoA) (Multivariate analysis-unsupervised ML)	Lower postprandial responses are related to alterations Proteobacteria and Enterobacteriaceae based on the ML algorithm.
Takahashi S et al., 2014 [81]	Case-control Study	Mice (C57BL/6J) 5 groups (*n* = 8–9), (9 weeks) Caffeine (2 g coffee powder/140 mL) -Normal diet group = 10% fat -High-fat diet group = 60% fat -Caffeinated coffee group = A high-fat diet + 2% caffeine -Decaffeinated coffee group = A high-fat diet + 2% decaffeinated coffee. -Green unroasted coffee group = A high-fat diet + 2% unroasted caffeinated coffee	Biochemical measurement: Hepatic lipid composition (the Folch method (Folch, Lees, & Sloane, 1957)), plasma liver enzymes (The transaminase C II-test WAKO kit) Genomics: (DNA microarray platform, Affymetrix) Proteomics: two-dimensional electrophoresis combined with MALDI-TOF mass spectrometry Metabolomics: Millipore Ultrafree-MC PLHCC HMT/CE-TOF-MS) analysis	PCA (Unsupervised ML)	Up-regulation of the iso-citrate dehydrogenase, lipid metabolism and ATP turnover were related anti-obesity effects of different types of coffee.

F, female; M, male; ANOVA, analysis of variance; GCM, general circulation model; CGM, continues glucose monitor; PERMANOVA, permutational multivariate analysis of variance; HPLC, high-performance liquid chromatography; LC-MS, liquid chromatography-mass spectrometry; NMR, nuclear magnetic resonance; LC-HRMS, liquid chromatography-high resolution mass spectrometry; HOMA-IR, homeostatic model assessment of insulin resistance; BLASTP, basic local alignment search tool; BMI, body mass index; CAMP, cyclic adenosine monophosphate; LFT, liver function test; NAFLD, non-alcoholic fatty liver disease; UPLC, ultra-performance liquid chromatography; DIABLO, Data Integration Analysis for Biomarker discovery using Latent variable approaches for ‘Omics studies; QIIME, quantitative insight into microbial ecology; CRP, c-reactive protein; ALT, alanine aminotransferase; AST, aspartate aminotransferase; WTP, diet containing whole grains, traditional medicinal foods, and probiotics; OPLS, orthogonal projections to latent structures discriminant analysis; MALDI-TOF, matrix-assisted laser desorption—ionisation-time of flight mass spectrometry; ATP, adenosine triphosphate; GC-TOF-MS, Gas chromatography with a time of flight mass spectrometer; PLS-DA, Partial least squares discriminant analysis; WGCNA, weighted correlation network analysis; KEGG, Kyoto Encyclopedia of Genes and Genomes; PLSR, partial least squares regression; PCoA, Principal coordinates analysis; PICRUST, predicted microbial metagenomes using a script; PCA, Principle component analysis; OPLS-DA, Orthogonal projection to latent structure-discriminant analysis; GC-MS, Gas chromatography coupled with mass spectrometry; CR, calorie restriction; BCAA, branched chain amino acid; HAMRS2, High-amylose-maize resistant starch type 2; CAF, diet involves feeding experimental animals a choice of human food items to stimulate energy intake (diet-induced thermogenesis).
